# Domains and Methods Used to Assess Home Telemonitoring Scalability: Systematic Review

**DOI:** 10.2196/29381

**Published:** 2021-08-19

**Authors:** Salome Azevedo, Teresa Cipriano Rodrigues, Ana Rita Londral

**Affiliations:** 1 Value for Health CoLAB NOVA Medical School Lisbon Portugal; 2 Comprehensive Health Research Centre NOVA Medical School, UNL Lisbon Portugal; 3 CEG-IST, Centre for Management Studies of Instituto Superior Técnico Universidade de Lisboa Lisbon Portugal

**Keywords:** telemonitoring, scalability, home telecare, systematic review

## Abstract

**Background:**

The COVID-19 pandemic catalyzed the adoption of home telemonitoring to cope with social distancing challenges. Recent research on home telemonitoring demonstrated benefits concerning the capacity, patient empowerment, and treatment commitment of health care systems. Moreover, for some diseases, it revealed significant improvement in clinical outcomes. Nevertheless, when policy makers and practitioners decide whether to scale-up a technology-based health intervention from a research study to mainstream care delivery, it is essential to assess other relevant domains, such as its feasibility to be expanded under real-world conditions. Therefore, scalability assessment is critical, and it encompasses multiple domains to ensure population-wide access to the benefits of the growing technological potential for home telemonitoring services in health care.

**Objective:**

This systematic review aims to identify the domains and methods used in peer-reviewed research studies that assess the scalability of home telemonitoring–based interventions under real-world conditions.

**Methods:**

The authors followed the PRISMA (Preferred Reporting Items for Systematic Reviews and Meta-Analysis) guidelines and used multiple databases (PubMed, Scopus, Web of Science, and EconLit). An integrative synthesis of the eligible studies was conducted to better explore each intervention and summarize relevant information concerning the target audience, intervention duration and setting, and type of technology. Each study design was classified based on the strength of its evidence. Lastly, the authors conducted narrative and thematic analyses to identify the domains, and qualitative and quantitative methods used to support scalability assessment.

**Results:**

This review evaluated 13 articles focusing on the potential of scaling up a home telemonitoring intervention. Most of the studies considered the following domains relevant for scalability assessment: problem (13), intervention (12), effectiveness (13), and costs and benefits (10). Although cost-effectiveness was the most common evaluation method, the authors identified seven additional cost analysis methods to evaluate the costs. Other domains were less considered, such as the sociopolitical context (2), workforce (4), and technological infrastructure (3). Researchers used different methodological approaches to assess the effectiveness, costs and benefits, fidelity, and acceptability.

**Conclusions:**

This systematic review suggests that when assessing scalability, researchers select the domains specifically related to the intervention while ignoring others related to the contextual, technological, and environmental factors, which are also relevant. Additionally, studies report using different methods to evaluate the same domain, which makes comparison difficult. Future work should address research on the minimum required domains to assess the scalability of remote telemonitoring services and suggest methods that allow comparison among studies to provide better support to decision makers during large-scale implementation.

## Introduction

The Universal Health Coverage commitment aligned with the emergence of COVID-19 reinforced the value of telemedicine services and elected these services crucial to coping with the pandemic’s challenges in the health care sector. Since the pandemic reached the western countries, home telemonitoring offered an alternative to control the health status of infected nonsevere patients at their homes to avoid unnecessary visits to the hospital [1].

During the early part of 2020, from a social perspective, the fast-track solution to prevent the spread of COVID-19 focused on social distancing [2]. Governments forced people to stay at home, canceled mass gatherings, imposed teleworking, and closed all educational institutions [3]. From a health care perspective, governments took some extreme measures to increase the capacity to cope with the virus, namely reduction or deferral of nonurgent care and hands-on visits, and postponement of nonurgent surgeries [4]. These measures exposed high-risk groups, such as the elderly at home, people at long-term care facilities, patients with chronic conditions, and hidden diseases [5]. Inevitably, physicians started following-up with their patients through video calls and remote monitoring to continue treatment and avoid long-term complications [6]. In parallel, health care providers launched new telehealth services to assist patients in their homes [7]. Policy makers and practitioners did not have enough information to decide which pilot intervention they should disseminate into real-world settings, considering different financial reimbursement strategies, health care system organizations, and workforce acceptance levels [8].

With technological progression and decreasing equipment costs, remote patient monitoring emerged as a telemedicine application. It comprises interactive and noninteractive technologies to support health care and monitor patients’ health status in their homes [9].

Home telemonitoring is one type of remote patient monitoring, which has shown and is showing potential to improve clinical and patient-reported outcomes and ensure cost reductions for health care practices [10]. In this work, the authors consider the definition given by Paré and colleagues [11] for home telemonitoring. A service based on home telemonitoring consists of health care professionals monitoring the patient's health status at a distance. Patients or caregivers transmit their health-related data to a responsible health care professional through information and telecommunication technologies. Research on home telemonitoring showed benefits concerning health care systems’ capacity constraints [12], patient empowerment, and treatment commitment [13]. It revealed significant improvement in clinical outcomes even in some diseases [11]. Despite the considerable investment in accelerating health information technology [14], there is not enough information on determining whether home telemonitoring is appropriate and feasible for implementation in a real-world context [15]. Scaling up a health intervention requires wise and efficient spending of resources [16]. Therefore, it is crucial to assess the suitability of scaling up home telemonitoring interventions with proven efficacy to provide answers to the following two questions [17]: *Does it work in practice? Is it worth it?*

To answer these questions and decide which technology-based health intervention can be scaled up for mainstream care delivery, one must assess its scalability (ie, the ability to be expanded to real-world conditions without compromising on effectiveness and access to the eligible population) [18].

Most of the studies focus only on assessing the effectiveness and costs of a health intervention. Nevertheless, these are two of many considerations to address when evaluating the potential of scaling up an intervention [19]. Other domains such as the feasibility and adaptability of the health intervention and the political or strategic contexts are rarely analyzed. As emphasized by Milat and his colleagues [15] in their recently proposed Intervention Scalability Assessment Tool (ISAT), assessing a health intervention’s scalability involves considering multiple domains, such as the political and strategic contexts, workforce, and infrastructure, among others.

There is a need to conduct evidence-based studies that assess pilot interventions’ potential to achieve population-wide benefits [20]. Scalability studies that also consider the intervention’s suitability to the socioeconomic context in question are important to estimate the success of deploying these interventions in different contexts [15].

Owing to the lack of research on scalability analysis, in this paper, the authors present a systematic review, based on Milat and colleagues’ domains [15], to identify and characterize methods used to assess the potential to scale-up home telemonitoring interventions in the context of a growing telehealth service in the industry. This study focuses on peer-reviewed studies conducted to evaluate the scalability of follow-up interventions based on home telemonitoring. The authors aim to provide a comprehensive overview of these studies concerning the domains and methods used and identify gaps for future research to address when evaluating the potential to implement or scale-up home telemonitoring interventions. As the authors are not aware of other systematic reviews focusing on this aspect, they believe that this review will enlighten researchers, practitioners, and policy makers regarding the most used strategies to assess the scalability of home telemonitoring interventions.

## Methods

The search strategy followed the PRISMA (Preferred Reporting Items for Systematic Reviews and Meta-Analysis) guidelines to conduct the review [21]. The population, intervention, comparison, outcome (PICO) framework [21] allowed the identification of key concepts such as “Home Telemonitoring,” “Follow-up,” “Scalability,” and “Assessment” to formulate a well-focused question and facilitate the literature search. To optimize the search through effective queries, the authors used PubMed’s Medical Subject Headings (MeSH) to identify indexed terms [22]. This step was fundamental as this review emerges from the combination of research fields with different terms for the same concept. Textbox 1 presents the rationale used to build the final query used in each database.

**Queries used to search each database.**

1. (((Telemonitoring) OR (Home remote monitoring)) AND (Mobile Health OR health OR mHealth OR eHealth OR Telehealth OR Telemedicine)) OR (Telehomecare)

2. (Scalability) OR (Feasibility) OR (Scaling up OR scale up OR upscale OR up-scale OR scale-up) OR ((Deployment OR Implementation OR Application) OR (Broad-scale OR Wide-scale OR Widespread OR Mainstream)) OR (((Efficienc*) AND (Program OR Intervention)) OR Economic* Viability)

3. (Follow-up Care* OR Follow Up Care* OR Care*) OR (Case Management OR Patient Care Planning)

4. ((Appraisal* OR Evaluation* OR Assessment* OR Appropriateness) AND ((Impact) OR (Cost-Effective* OR Qualitative OR Quantitative OR Index* OR Methodolog*) OR (Clinical Trial* AND (Pragmatic OR Naturalistic Randomized OR Practical OR Real World)) OR (Sustainability) OR (Profitability) OR (Risk*)))

5. #1 AND #2 AND #3 AND #4

**Figure 1:**
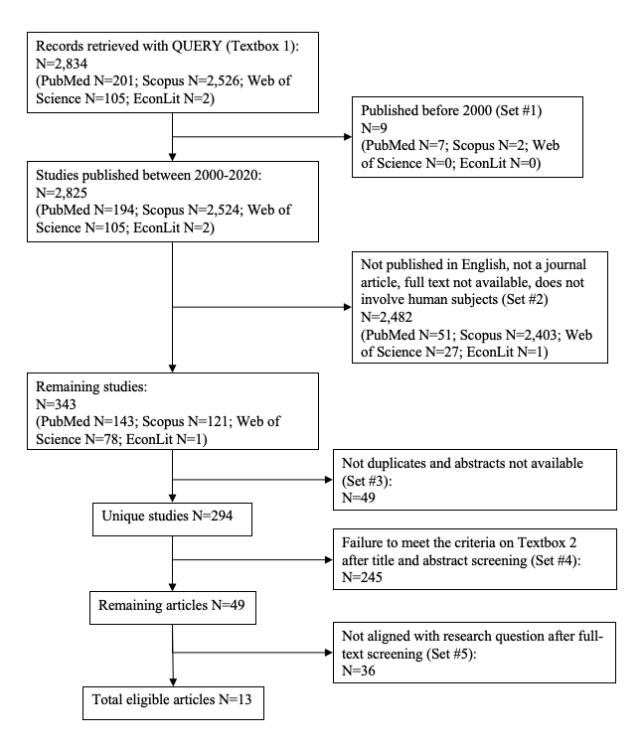
PRISMA (Preferred Reporting Items for Systematic Reviews and Meta-Analyses) flow diagram showing the included studies.

Figure 1 illustrates the search performed in PubMed, Scopus, Web of Science, and EconLit covering studies from 2000 to 2020 (Figure 1 - Set #1). The authors chose to explore EconLit owing to the economic evaluation required to assess a health care intervention’s scalability. The authors selected full-text and peer-reviewed papers written in English (Figure 1 - Set #2).

After removing the duplicates and references without abstracts (Figure 1 - Set #3), two authors independently scanned the titles and abstracts identified in the literature search and applied the selection criteria presented in Textbox 2 (Figure 1 - Set #4).

To guarantee that the article’s topic aligned with the research question, the same authors scanned the 49 full-text articles, which reduced the number of studies considered for review to 13 (Set #5).

The authors analyzed 13 full-text articles, corresponding to 13 studies, in detail and registered all the observations in a literature matrix [23]. First, to better explore each intervention and summarize relevant, well-specified, and secure data, the authors conducted an integrative synthesis. The main variables were the country of origin, publication year, sample size, setting, duration of follow-up, comparator arms, type of technology, and study outcomes [24].

Second, the authors assessed the strength of each eligible study’s evidence according to the 9-level classification system proposed by Jovell and Navarro-Rubio [25].

**Eligibility criteria for screening titles, abstracts, and full-text papers.**

**Inclusion criteria**

- Health interventions shown to be efficacious on a small scale or under controlled conditions
- Assessment of the health intervention’s ability to be expanded to real-world conditions to reach a more significant proportion of the eligible population while retaining effectiveness.
- Studies assessing at least one domain of scalability through the evaluation of feasibility, acceptability, costs, sustainability or, adaptability (ie, to suit the needs of the context in which it is to be scaled up)
- Described methods to assess the scalability of a health intervention

**Exclusion criteria**

- Telemonitoring involving invasive medical devices
- Studies that use telemonitoring “not involving the patients, their relatives, or informal caregivers, their relatives, or informal caregivers
- Studies that described the concept of scalability without providing an assessment method
- Studies just focusing on describing disease risk patterns or intervention efficacy testing
- Study protocols or medical testing procedures for potential scalability assessment and possible scale-up
- Statistical or conceptual modeling without a real-world study
- Facilitators and barriers to scale-up within specific interventions or general experiences of scale-up that did not provide a scalability assessment method
- Studies recommending an assessment method (of feasibility or acceptability or costs or sustainability or adaptability), but that did not assess the potential to scale-up a telemonitoring-based health intervention

Finally, they conducted narrative and thematic analyses to identify themes and patterns in the eligible articles and outline the findings under specific headings [24] to better examine how each study assessed the potential of scaling up an intervention. When disagreements occurred, the authors reached a consensus via discussion. One author extracted data from the studies and completed quotes, and the second author validated the data according to the definition of each category. The authors conducted this analysis based on the work undertaken by Milat and colleagues [18] in the development of a tool to perform systematic assessments of the suitability of health interventions for scale-up (ISAT). ISAT comprises three parts: setting the scene, planning the intervention implementation, and summarizing the scalability assessment. The first two parts made it possible to classify each study according to the stage of scale-up, context, and focus area. Moreover, Milat and colleagues’ domains enabled the authors to identify the methods and instruments used by the researchers to assess the intervention’s scalability [18].

The research conducted for each domain assessed in the eligible papers was classified as qualitative or quantitative. The research was classified as qualitative if it was based on the description of experiences, emotions, behaviors, events, or actions [26] and quantitative when the respective authors used numerical data to measure, categorize, or identify patterns, relationships, or generalizations through statistical analysis [26].

## Results

### Country of Origin and Year of Publication

From 2009 to 2020, the authors analyzed 13 studies in 7 countries, which focused on the potential to scale-up home telemonitoring health care interventions; however, more than half (n=7) were published between 2018 and 2020. Most of the articles (n=8) were from Canada and the United States, whereas the rest were from 5 European countries—Denmark (n=1), Italy (n=1), Lithuania (n=1), Netherlands (n=1), and Spain (n=1).

### Population and Home Telemonitoring Intervention Assessment

#### Target Condition or Disease

The studies addressed either chronic or acute conditions, with a higher number of studies addressing only chronic conditions (n=8). The full spectrum of chronic conditions covered were cardiovascular diseases (n=4), chronic obstructive pulmonary diseases (n=2), cerebrovascular diseases (n=1), chronic obstructive sleep apnea (n=1), cystic fibrosis (n=1), and diabetes mellitus (gestational [n=1] and type 1 and 2 [n=1]). Further, one study only characterized the patients’ condition as chronic or acute, and the remaining studies addressed multiple conditions (eg, surgical patients, cardiovascular and pulmonary diseases, diabetes mellitus).

#### Duration and Setting of Home Telemonitoring Intervention

Home telemonitoring was integrated into a follow-up service in the 13 studies and required a responsible health care professional (or a team) to manage the patient’s care. The minimum duration of the follow-up was 3 consecutive nights (sleep apnea [27]). However, the 1-year (n=4) and 6-month (n=4) follow-up interventions were the most implemented. In particular, authors reporting the secondary prevention of cerebrovascular disease [28] defined the intervention according to recommended monitoring protocols, assuming a 20-year time horizon for the modeling strategy. Moreover, 10 studies had 2 dedicated teams for executing the intervention; one was responsible for the patient’s holistic care management and the other for telecare management. In two studies, the conventional care team was accountable for usual care and telecare management, and in the other, there was no traditional care team.

#### Types of Technologies

The technologies used in the studies ranged from a kit with just one regular telephone (1) to an integrated communication and data collection system with mobile devices (5). Moreover, six studies conducted home telemonitoring interventions with an integrated clinical data system, remote monitoring digital technology (mobile devices that collect physiological signs), and a telephone.

### Study Design Assessment

#### Study Characteristics

The average total sample size of the studies was approximately 436 (maximum: 3086, minimum: 34), with an average treatment and control group size of 260.

To better understand the type of research conducted, it is essential to highlight that 6 out of the 13 studies were experimental. Therefore, the authors of these studies allocated participants to different treatment groups. As the other 7 studies were observational, there was no allocation of the participants. Most of the studies (n=10) were comparative studies (control group) with conventional care services, and the other 3 were single-arm studies.

#### Study Design Classification

According to the 9-level classification system proposed by Jovell and Navarro-Rubio [25], the studies conducted by Padwal and colleagues [28], and Vestergaard and colleagues [29] were classified as “very good,” as they conducted randomized controlled trials with large samples. The studies by Lugo and colleagues [27], and Paré and colleagues [30] were classified as “good” as these studies were randomized controlled trials with small samples. Furthermore, the studies of Ware and colleagues [31], as well as Zaliūnas and colleagues [32], were classified as “poor” because they consisted of noncontrolled clinical series or descriptive studies. The other 7 were classified as fair and included nonrandomized controlled prospective studies (n=3), cohort studies (n=3), and case-control studies (n=1).

### Scalability Assessment

Table 1 displays the scalability assessment domains for each study.

**Table 1:** Scalability assessment domains for each study.

Application field	Stage of scale-up	Domains for scale-up						Domains for implementation planning				
		Problem	Intervention	Context	Effectiveness	Costs and benefits	Fidelity and adaptability		Reach and acceptability	Setting and workforce	Infrastructure	Sustainability
Improved health outcomes in a rural area [33]	Pre–scale-up	Yes	Yes	Yes	Yes	Yes	No		Yes	No	No	No
Diabetes [34]	Pre–scale-up	Yes	Yes	Yes	Yes	Yes	Yes		Yes	Yes	Yes	Yes
Cystic fibrosis [35]	Pre–scale-up	Yes	Yes	No	Yes	Yes	No		No	Yes	Yes	Yes
Chronic heart failure [36]	Pre–scale-up	Yes	No	No	Yes	Yes	No		No	No	No	No
Obstructive sleep apnea [27]	Pre–scale-up	Yes	Yes	No	Yes	Yes	Yes		Yes	Yes	Yes	Yes
Secondary prevention of cerebrovascular disease [28]	Pre–scale-up	Yes	Yes	Yes	Yes	Yes	Yes		No	No	No	Yes
Heart failure [29]	Pre–scale-up	Yes	Yes	Yes	Yes	Yes	No		No	No	No	No
Gestational diabetes mellitus [37]	Pre–scale-up	Yes	Yes	Yes	Yes	Yes	Yes		Yes	Yes	Yes	Yes
Rural home health agencies [38]	Scale-up	Yes	Yes	Yes	Yes	Yes	Yes		Yes	No	Yes	Yes
Chronic obstructive pulmonary disease [30]	Scale-up	Yes	Yes	Yes	Yes	Yes	No		Yes	Yes	Yes	Yes
Ischemic heart disease [32]	Implementation	Yes	Yes	Yes	Yes	No	Yes		Yes	Yes	Yes	Yes
Heart failure [31]	Implementation	Yes	Yes	Yes	Yes	No	Yes		Yes	Yes	Yes	Yes
Chronic obstructive pulmonary disease [39]	Implementation	Yes	Yes	Yes	Yes	No	No		Yes	Yes	Yes	Yes

#### Scale-Up Stages

The authors classified eight studies as being in the pre–scale-up stage because their descriptions consisted of steps or activities conducted before scaling up the evidence-based home telemonitoring intervention. Two studies described steps or actions involved in the dissemination of the intervention. The authors classified the other three studies as being in the implementation stage because their descriptions indicated using or integrating the evidence-based intervention within a setting.

#### Domains Considered for Scale-Up

Although all the studies described the problem under intervention and the target population, one study [36] did not provide details concerning the proposed home telemonitoring intervention to address the issue. All studies referred to the level of evidence available to support the proposed intervention’s scale-up, either by referring to their work or other scientific literature., Three studies did not consider the known costs and benefits of delivering the intervention [31,32,39], and three more did not consider the strategic/political/environmental contexts that influence the scaling up of the intervention [27,35,36].

#### Domains Considered for Implementation Planning

Seven studies considered intervention changes when assessing fidelity, and nine studies assessed the level of acceptability perceived by the program deliverers or recipients of the intervention. Further, 9 studies referred to the definition of the intervention settings and the workforce required to scale-up, and 10 described the necessary infrastructure.

All the studies accounted for the sustainability of the home telemonitoring service by either referring to the long-term outcomes of the scale-up or the medium- and long-term sustainability of the intervention following scale-up.

#### Methods for Scalability Assessment

This section explains the research foci and methods used by the eligible studies in each domain of scalability assessment. When describing the problems, interventions, and contexts of their studies, all the researchers adopted qualitative research methods, as Table 2 shows. The definitions of the domains and research foci are given in Multimedia Appendix 1. We have included six publications [40-45] in this appendix.

**Table 2:** Qualitative studies on scalability assessment considering the problem, intervention, and context domains for scale-up.

Domain	Research focus	Research type	Data collection technique	Data analysis technique	Studies, n	Reference
Problem	Problem description	Qualitative	Document analysis	Narrative summary	13	[27-39,46-50]
Intervention	Intervention description	Qualitative	Document analysis	Narrative summary	12	[27-35,37-39]
Context	Context description	Qualitative	Document analysis	Narrative summary	10	[28-34,37-39]

All the studies adopted quantitative research methods to assess clinical outcomes namely surveys or questionnaires (n=10), published databases (n=2), and observations (n=1) (Table 3). To assess humanistic and satisfaction outcomes, the researchers chose surveys or questionnaires; however, for assessing for usage outcomes, they either conducted observations (n=9) or used published databases (n=3). As for validated instruments, only one was used in one study [27] to assess clinical outcomes, namely the Epworth Sleepiness Scale (ESS) [51]. For assessing humanistic outcomes, three validated questionnaires were used: EuroQol 5-Dimensions 5-Levels (EQ-5D-5L) [52] in the contexts of heart failure [29] and obstructive sleep apnea [27]; Quebec Sleep Questionnaire (QSQ) [53] for obstructive sleep apnea; and Chronic obstructive pulmonary disease Assessment Test (CAT) [54] for chronic obstructive pulmonary disease [39]. In the context of ischemic heart disease [32], two more validated questionnaires were used: Patient Satisfaction Questionnaire Form III (PSQIII) [55] and Thought Control Questionnaire (TCQ) [56].

**Table 3:** Quantitative research studies involving data analyses using descriptive and inferential statistics for scalability assessment considering the effectiveness domain for scale-up.

Research focus and data collection technique		Studies, n	Reference
**Clinical outcome assessment**			
	Observation; published databases	3	[28,36,38]
	Nonvalidated surveys or questionnaires	9	[29-35,37,39]
	Validated surveys or questionnaires	1	[27]
**Humanistic outcome assessment**			
	Nonvalidated surveys or questionnaires	3	[32-34]
	Validated surveys or questionnaires	3	[27,29,39]
**Satisfaction assessment**			
	Nonvalidated surveys or questionnaires	7	[27,29,30,33,34,37,39]
	Validated surveys or questionnaires	1	[32]

For the domains of fidelity and acceptability, quantitative research methods involving observations were more predominantly used as the main data collection methods, as shown in Tables 4 and 5. Contrarily, for analyzing infrastructure, setting, and workforce, most of the studies chose qualitative techniques (n=8).

**Table 4:** Studies on scalability assessment concerning the reach and acceptability domain for implementation planning involving data analyses using descriptive and inferential statistics.

Research focus and type		Data collection technique		Studies, n		Reference
**Acceptability assessment**						
	Quantitative	Observation	7		[27,30,32-34,37,39]	
	Qualitative	Semistructured interviews	1		[38]	
**Compliance assessment**						
	Quantitative	Nonvalidated surveys or questionnaires	1		[31]	
	Quantitative	Validated surveys or questionnaires	1		[32]	
**Penetration assessment**						
	Quantitative	Observation	2		[31,37]	

**Table 5:** Research focus and methods found in the studies for scalability assessment concerning the fidelity and adaptability domain for implementation planning.

Research focus and type			Data collection technique		Data analysis technique		Studies, n		Reference
**Adaptability assessment**									
	Quantitative	Observation		Descriptive statistics; inferential statistics		1		[27]	
	Qualitative	Observations; oral history or life stories		Narrative summary		2		[32,38]	
**Feasibility assessment**									
	Quantitative	Observation		Descriptive statistics; inferential statistics		2		[31,37]	

When conducting economic evaluation (Table 6), the authors found 7 different types of techniques used across 10 studies (see Multimedia Appendix 2 for the main results of the studies that conducted economic evaluation of home telemonitoring). The most popular technique was cost-effectiveness analysis used in three studies with different fields of application. These three studies were able to show outcome improvements and cost savings. Table 7 presents the scalability assessment studies concerning the setting and workforce, infrastructure, and sustainability domains for implementation planning

**Table 6:** Quantitative research studies focusing on data collection using document screening and published databases for scalability assessment considering the costs and benefits domain for scale-up (research focus: economic evaluation).

Data analysis technique	Studies, n	Reference
Cost analysis	2	[34,35]
Cost-benefit	1	[38]
Cost-effectiveness	3	[27,33,37]
Cost minimization	1	[30]
Cost utility	2	[28,29]
Cost-saving simulation	1	[35]
Value of information analysis	1	[36]

**Table 7:** Studies on scalability assessment concerning the setting and workforce, infrastructure, and sustainability domains for implementation planning.

Domain and research focus and type				Data collection technique		Data analysis technique		Studies, n		Reference
**Setting and workforce**										
	**Setting and workforce assessment**									
		Qualitative	Observations; oral history or life stories		Narrative summary		8		[27,28,31,32,34,35,37,39]	
		Quantitative	Observation		Descriptive statistics		1		[30]	
**Infrastructure**										
	**Infrastructure assessment**									
		Qualitative	Observations; oral history or life stories		Narrative summary		9		[27,28,30,32,34,35,37-39]	
		Qualitative	Semistructured Interviews		Descriptive statistics		1		[31]	
**Sustainability**										
	**Opportunity and challenge assessment**									
		Qualitative	Observations; oral history or life stories		Narrative summary		12		[27-30,32-39]	
		Qualitative	Semistructured interviews		Narrative summary		1		[31]	

#### Scalability Assessment

All the 13 articles assessed scalability based on the results achieved in the respective studies. Table 8 summarizes the assessments obtained through narrative analysis. On the one hand, two studies provided positive assessments regarding the potential to scale-up the intervention. On the other hand, eight studies highlighted the need for cost-effectiveness or cost-benefit analysis before proceeding to scale-up the intervention.

**Table 8:** Scalability assessment based on the authors’ conclusions in each study.

Scalability assessment	Studies, N	Reference
*Not* able to be expanded	1	[27]
Able to be expanded, *but* the diffusion and sustainability will depend on a supportive policy environment	1	[34]
Able to be expanded *but* requires cost-benefit analysis for reimbursement planning	3	[28,36,38,39]
Able to be expanded *but* requires cost-effectiveness analysis	3	[29,30,35]
Able to be expanded *but* requires some technical changes, cost-benefit analysis for reimbursement planning, and solutions for regulatory issues	2	[32,33]
Able to be expanded under real-world conditions	2	[31,37]

## Discussion

### Principal Results

Despite the rapid growth of telemedicine applications in the last few years, particularly after the emergence of COVID-19, scientific studies assessing the scalability of these health interventions are scarce [19].

In this review, all the eligible studies are from developed countries, particularly the United States and Canada. The absence of such studies in developing countries could be owing to the lack of specialized human resources, information and communications technology (ICT) infrastructure, and equipment [46]. Besides, the significant difference found between North America and Europe might be related to the requirement of evidence to justify private payer reimbursement for health care interventions [47] or the investment in developing strategies to encourage telemedicine adoption [48]. Nevertheless, this review has not identified studies from countries that invested significantly in telehealth solutions, such as the United Kingdom or Australia [46]. The justification for this might be the frequent research focus of health interventions on clinical effectiveness [11], instead of assessing their scale-up potential. More than half of the studies were published between 2018 and 2020. Thus, this research area is receiving more attention from the scientific community as a logical next step after demonstrating robust evidence regarding the effectiveness and technological maturity of such interventions.

The use of one of the most recent scalability assessment frameworks [18] granted the opportunity to compare the strategies used to assess the scale-up potentials of interventions in each study. This advantage of this framework is that it allows the analysis of different domains considering the stage of the transference process of an intervention from a research setting into the practical implementation stage.

This review suggests an agreement in some analyzed domains, such as problems, interventions, effectiveness, costs, and benefits, to support the decision to scale-up interventions. However, this is not the case for the methods and instruments used. For example, although cost-effectiveness was the most common approach across the 13 studies, researchers used 7 different cost analysis methods. Moreover, to demonstrate effectiveness, studies provided evidence of different outcomes, such as clinical, humanistic, and utilization outcomes. This inconsistency leads to different scalability assessments and does not enable comparing interventions with home telemonitoring technologies.

There is a recognized methodological gap in understanding other relevant domains such as the sociopolitical context, setting, workforce, and implementation infrastructure to provide the home telemonitoring intervention to the target population. A common framework will allow determining if interventions demonstrated as effective are appropriate and feasible in other settings [18,49].

Lastly, another relevant result obtained from this systematic review was that researchers assigned different weights to the analyzed domains when concluding the intervention scalability. On the one hand, 12 studies concluded their ability to scale- up based on the costs and outcomes of the interventions, although they had analyzed other domains. On the other hand, one study restrained the decision to scale-up the intervention based on the policy environment. Future research should address the influence that each domain has on the final decision to scale-up the interventions with sound and transparent methods, avoiding mistakes reported in the literature [50].

### Limitations

This relevant limitation of this review might be associated with the low maturity of this research area, despite its recent growth. Additionally, one database filter concerned peer-reviewed journals, which influenced the rejection of studies with no statistical significance but could have been relevant in this review with respect to the domains and methods used when assessing scalability. This review only considered studies published in English, which might have influenced the number of eligible studies. Moreover, the authors did not conduct a meta-analysis owing to the limited number of studies on this subject. Finally, the domains used to analyze the scalability assessment strategies were predefined, thus limiting the spectrum of domains studied.

### Conclusions

Studies on home telemonitoring interventions integrated into follow-up care have already proved their efficacy. Although some studies focused on including domains such as effectiveness, costs, and benefits, these are not enough to assess the potential of scaling up these interventions. As technology progresses and the need for providing care to more people in their homes increases, it is extremely important to conduct more studies on scalability assessment considering domains such as workforce and infrastructure characteristics and the strategic context. Future research should establish rigorous study designs and scientific methods to assess scalability based on the results of this systematic review. Further understanding of the usage of health services and medium- and long-term sustainability of interventions would yield more robust evidence to support their future integration into mainstream care delivery systems. This research area, although still emerging, will advance knowledge on the factors that influence the successful scale-up of interventions.

## Appendix

Multimedia Appendix 1 Glossary of research methods and scalability assessment domains used to systematically review the eligible studies in this work.

Multimedia Appendix 2 Main results of the economic evaluations conducted in each eligible study that addressed the domains of costs and benefits.

## Abbreviations

CAT: Chronic Obstructive Pulmonary Disease Assessment Test
EQ-5D-5L: EuroQol 5-Dimensions 5-Levels
ESS: Epworth Sleepiness Scale
ICT: information and communications technology
ISAT: Intervention Scalability Assessment Tool
MeSH: Medical Subject Heading
PRISMA: Preferred Reporting Items for Systematic Reviews and Meta-Analysis
PSQIII: Patient Satisfaction Questionnaire Form III
QSQ: Quebec Sleep Questionnaire
TCQ: Thought Control Questionnaire
