# Supplementary material for: Domains and Methods Used to Assess Home Telemonitoring Scalability: Systematic Review
Source: JMIR Mhealth Uhealth. 2021 Aug 19;9(8):e29381. doi: 10.2196/29381 (PMC8414303; doi:10.2196/29381)
Supplement: Multimedia Appendix 1 [file mhealth_v9i8e29381_app1.pdf]

## Multimedia Appendix

Glossary of scalability assessment domains and research methods used to review the eligible studies in this review systematically.

| **Concept** | **Description** | **Reference** |
|---|---|---|
| Problem | Description of the problem being addressed, who it affects, what it involves, and how it is currently being addressed. | [18] |
| Intervention | Description of the proposed programme or intervention to address the problem. | [18] |
| Strategic/political context | Description of the strategic, political, or environmental contextual factors that potentially influence any intervention to be scaled up. | [18] |
| Evidence of Effectiveness | Level of evidence available to support the scale-up of the proposed intervention, such as scientific literature and other known evaluations of the intervention. | [18] |
| Intervention Costs & Benefits | Consideration of the known costs of the intervention delivery and any quantifiable benefits. This includes the results of any types of economic evaluation studies. | [18] |
| Fidelity & Adaptation | Description of the proposed changes to the intervention required for scale-up. | [18] |
| Reach & Acceptability | Description of the possible reach and acceptability of the intervention for the target population. | [18] |
| Delivery Setting & Workforce | Define the setting within which the intervention is delivered as well as the delivery workforce. | [18] |
| Implementation Infrastructure | Implementation infrastructure is required for scale-up. | [18] |
| Sustainability | Longer-term outcomes of the scale-up and how once scaled up, the intervention could be sustainable over the medium to longer term. | [18] |

| | | |
|---|---|---|
| Clinical Outcomes Assessment | Clinical outcomes assessment refers to the evaluation of efficacy endpoints when developing a therapy for a disease or condition. The patient assessment used in an endpoint, is the measuring instrument that provides a rating or score (categorical or continuous) that is intended to represent some aspect of the patient's health status. | [45] |
| Humanistic Outcomes Assessment | Humanistic outcomes assessment refers to the evaluation of outcomes of interest to patients, which are clinically meaningful with practical implications for disease recognition and management and that are based on a patient's unique perspective, e.g., patient-reported scales that indicate pain level, degree of functioning, etc. This category of outcomes involves measures of health-related quality of life (HRQoL) and patient-reported outcomes measurements, which include measures of HRQoL. | [46] |
| Utilization Outcomes Assessment | Utilization outcomes measurement refers to the evaluation of measures of health resource utilization, such as number of inpatient or outpatient visits, total days of hospitalization in a given year, or number of days treated with antibiotics. These outcomes are often used as proxies for measuring cost. | [46] |
| Satisfaction Assessment | Assessment of patient' and healthcare professionals' satisfaction with the health intervention. | - |
| Economic Evaluation | Economic evaluation involves a comparison between alternative strategies, considering both their costs and their benefits. | [47] |
| Fidelity Assessment | Fidelity assessment refers to the confirmation that the manipulation of the independent variable occurred as planned to ensure that fair, powerful, and valid comparisons of replicable treatments can be made. | [48] |
| Feasibility Assessment | Feasibility assessment consists of determining whether an intervention should be adjusted to be relevant and sustainable through the identification of aspects requiring modification. | [49] |
| Adaptability Assessment | Proposed changes to the intervention required for scale-up. | [18] |
| Acceptability Assessment | Acceptability assessment refers to the evaluation of the level of satisfaction, suitability or attractiveness perceived by the programme delivers or recipients. | [49] |

| | | |
|---|---|---|
| Adoption Assessment | Assessment of the number of adopters of the proposed programme or intervention. | [30] |
| Compliance Assessment | Compliance assessment refers to the evaluation of the extent to which the patient follows the prescribed and dispensed regimen as intended by the prescriber and dispenser. | [50] |
| Penetration Assessment | Assessment of the level of integration of the programme or intervention within the existing services. | [30] |

This is a Multimedia Appendix to a full manuscript published in the J Med Internet Res. For full copyright and citation information see http://dx.doi.org/10.2196/jmir.29381
