# Supplementary material for: Domains and Methods Used to Assess Home Telemonitoring Scalability: Systematic Review
Source: JMIR Mhealth Uhealth. 2021 Aug 19;9(8):e29381. doi: 10.2196/29381 (PMC8414303; doi:10.2196/29381)
Supplement: Multimedia Appendix 2 [file mhealth_v9i8e29381_app2.pdf]

Multimedia Appendix

Main results of the economic evaluations conducted in each eligible study that addressed the domain of costs and benefits.

Study	Telemonitoring (n1); Control (n2)	Type of Analysis	Main Results
Rural home health agencies [42]	1513; 1573	Cost-Benefit Analysis	Average number of home health episodes per patient, the length of care, and the mean number of visits per episode was lower in the telemonitoring group. The total average cost/episode was lower in the control group.
Secondary prevention of cerebrovascular disease [26]	228; 222	Cost-Utility Analysis	Telemonitoring resulted in an incremental 0.83 QALYs and cost savings of $1929 compared to control. Telemonitoring was the preferred treatment in 73% of simulations at a willingness to pay (WTP) of $0 per QALY, and 99% of the ICURs fall below a WTP threshold of $10 000 per QALY.
Improve health outcomes in a rural area [37]	47; 47	Cost-Effectiveness Analysis	Telemonitoring resulted in cost reductions in personnel, traveling, and hospitalizations. Telemonitoring resulted in fewer hospital admissions (18 versus 23 admissions), shorter average lengths of stay (6.1 versus 7.6), and rehospitalizations (11 versus 16). The net difference in costs associated with telemonitoring was $106,601 during this 6-month period.
Chronic heart failure [40]	341;85	Value of Information Analysis	The cost of uncertainty regarding the decision on reimbursement of telehealth interventions for chronic heart failure patients is high in the Netherlands, and that future research is needed on the transition probabilities.
Heart failure [27]	134; 140	Cost-Utility Analysis; Cost-Effectiveness Analysis	Telemonitoring provided a positive incremental NMB of £5164. The 1-year adjusted QALY difference between the telemonitoring solution and the usual care group was 0.0034 (95% CI: −0.0711 to 0.0780). The adjusted difference in costs was -£5096 (95% CI: −8736 to −1456) corresponding to a reduction in total healthcare costs by 35%.
Cystic Fibrosis [39]	29; 25	Cost Analysis;	Potential saving of €40,397.00 per patient for 10 years, actualized at €36,802.97 for the follow-up of all patients enrolled.

		Costs-Saving Simulation	
Gestational Diabetes Mellitus [41]	80; 81	Cost-Effectiveness Analysis	A decrease in medical visits by 56% ($p < 0.001$) in the telemonitoring group. No difference between the two groups in diabetes control or maternal and fetal complications.<br>A 10-fold increase in nursing interventions in telemonitoring group.<br>Satisfaction with care was high.<br>Direct cost analysis revealed a savings of 16% in patients followed by telemonitoring.
Obstructive sleep apnoea [28]	94; 92	Cost-Effectiveness Analysis	The QSQ social interactions domain improved significantly more in the control group. The EQ-VAS improved more in the telemonitoring group.<br>Total costs were lower in the telemonitoring group.<br>Virtual Sleep Unit was cost-effective for a wide range of willingness to pay for QALYs.
Diabetes [38]	74; 274	Cost Analysis	Improvements in glycaemic control ($p = 0.01$) and patients' satisfaction with overall care ($p = 0.04$).<br>Telemonitoring reduced costs, yielding a net cost savings of 8.8%.
Chronic obstructive pulmonary [29]	60; 60	Cost-Minimization Analysis	Telemonitoring reduced the number of hospitalization days and the number of emergency room visits.<br>Telemonitoring saved $1613 per patient per year compared to traditional care, representing a net gain of 14%.

This is a Multimedia Appendix to a full manuscript published in the J Med Internet Res. For full copyright and citation information see http://dx.doi.org/10.2196/jmir.29381
